# Adjunctive Treatment With Eslicarbazepine Acetate for Adults and Children With Focal-Onset Epilepsy: A Meta-Analysis

**DOI:** 10.3389/fneur.2022.909471

**Published:** 2022-07-15

**Authors:** Yanqing Fei, Ruting Shi, Zhi Song

**Affiliations:** ^1^Department of Neurology, Third Xiangya Hospital, Central South University, Changsha, China; ^2^Department of Rehabilitation, Third Xiangya Hospital, Central South University, Changsha, China

**Keywords:** eslicarbazepine acetate, focal-onset epilepsy, adjunctive treatment, randomized controlled trials, meta-analysis

## Abstract

**Background:**

The efficacy and tolerability of eslicarbazepine acetate (ESL) in adults and children with focal-onset epilepsy (FOE) according to the dose remain to be validated. A meta-analysis based on randomized controlled trials (RCTs) was therefore conducted as a summary.

**Methods:**

Relevant RCTs were collected by systematic searching the electronic databases of PubMed, Cochrane's Library, Embase, Wanfang and CNKI from inception to May 16, 2022. The random-effect model was adopted to pool the results by incorporating the possible heterogeneity. Efficacy outcomes including responsive rate and effective rate, defined as cases with 50 and ≥75% reduction in seizure frequency compared to baseline, were determined, respectively. Incidence of severe adverse events (AE) leading to drug discontinuation was also evaluated.

**Results:**

Ten studies including 2,565 people with epilepsy contributed to the meta-analysis. For adults, ESL 400 mg/d did not improve the response rate or the effective rate; ESL 800 mg/d was associated with improved response rate (odds ratio [OR] 2.16, 95% confidence interval [CI]: 1.65–2.83, *p* < 0.001) and effective rate (OR 2.16, 95% CI: 1.41–3.30, *p* < 0.001) without significantly increased severe AE (OR 1.58, 95% CI: 0.90–2.78, *p* = 0.11); ESL 1,200 mg/d improved response rate (OR 2.49, *p* < 0.001) and effective rate (OR 3.09, *p* = 0.04), but significantly increased severe AE (OR 3.72, *p* < 0.001). For children, ESL also did not significantly improve the response rate (OR 1.76, *p* = 0.22) or the effective rate (OR 2.17, *p* = 0.13).

**Conclusion:**

ESL 800 mg/d is effective and well-tolerated as adjuvants for adults with FOE. Efficacy of ESL in children with FOE should be further evaluated.

## Introduction

Epilepsy is a common neurological disorder of global population of all ages (1–3). Although many regimens of anti-seizure medications (ASMs) have been developed and applied, ~30% of people with epilepsy still suffer from seizures (4). The recurrent episodes of seizures significantly threaten the health of people with epilepsy (5). Moreover, long-term recurrence of seizures despite of the use of multiple ASMs could adversely affect the quality of life and lead to the increased risk of affective disorders and neurocognitive dysfunction in people with epilepsy (6–8). Focal-onset epilepsy (FOE) is the most prevalent type of seizures and can pose a significant risk to individual health and impair quality of life, particularly when they are associated with impairment of awareness (9). Although the prognosis of people with FOE is generally good, symptoms of seizures are poorly controlled in some people of FOE despite of the allocation of routine ASMs (10). Eslicarbazepine acetate (ESL) is a blocker of the voltage-gated sodium channels, which has been suggested to be effective for people with refractory FOE (11). However, the findings were mainly derived from adult people with FOE of various doses of ESL (12). In view of some recent published studies regarding the role of ESL as an adjunctive treatment for people with refractory FOE (13–17), particularly for children with FOE (13, 16), we aimed to perform a meta-analysis to summarize the efficacy and tolerability of ESL for people with refractory FOE, in adults and children according to the dose of the drug.

## Methods

The PRISMA 2020 (Preferred Reporting Items for Systematic Reviews and Meta-Analyses 2020) statement (18, 19) was followed in the designing and implementation of the meta-analysis. The methods for analyses and reporting were in accordance with the instructions of the Cochrane Handbook guidelines (20).

### Database Search

PubMed, Cochrane Library (Cochrane Center Register of Controlled Trials), Embase, Wanfang and CNKI (China National Knowledge Infrastructure) were searched for relevant studies with a combined keywords of “eslicarbazepine” AND (“seizure” OR “epilepsia” OR “epilepsy”) AND (“randomized” OR “randomized” OR “randomly” OR “random” OR “placebo” OR “allocated” OR “control”) from inception of the database to May 16, 2022. Only clinical studies were considered. No restriction was applied to the language of publication. The citations of related reviews and original articles were also analyzed manually for possible relevant RCTs.

### Study Inclusion and Exclusion Criteria

Studies fulfilling the following criteria, as designed according to the PICOS criteria, were included for the meta-analysis.

- P (patients): adult or children with refractory or uncontrolled FOE;
- I (intervention): adjunctive treatment with ESL on the basis of routinely used ASMs;
- C (comparison): placebo on the basis of routinely used ASMs or routinely used ASMs only;
- O (outcomes): reporting at least one of the efficacy/safety outcomes. The efficacy outcomes included responsive rate and effective rate, defined as cases with ≥50 and ≥75% reduction in seizure frequency compared to baseline. The safety outcomes included the incidence of any adverse events (AE) and the severe AE leading to drug discontinuation, which were defined and judged by the criteria used in the original studies.
- S (study design): RCTs published as full-length peer-reviewed articles;

The exclusion criteria included the following.

- Not RCTs;
- Studies did not include people with FOE;
- Studies did not use ESL as an adjunctive treatment based on routinely used ASMs;
- Studies did not report the outcomes of interest;
- Studies published as conference abstracts.

### Data Collection and Quality Assessment

Two authors independently completed database search, data collection, and quality assessment. If disagreement occurred, consensus by discussion with the corresponding author is indicated as a solution. We collected data regarding information of study, design (blind or open-label), number of participants and the diagnosis, regimens of ESL treatment and controls, background therapy, treatment duration, and outcomes reported. Quality evaluation was achieved using the Cochrane's Risk of Bias Tool (20) according to the following aspects: (1) random sequence generation; (2) allocation concealment; (3) blinding of participants and personnel; (4) blinding of outcome assessors; (5) incomplete outcome data; (6) selective outcome reporting; and (7) other potential bias.

### Statistics

Incidence of the efficacy or the safety outcomes was separately evaluated via the odds ratios (ORs) and their 95% confidence intervals (CIs) in this meta-analysis. We used the Cochrane's Q test to detect the heterogeneity (21). For studies including multiple dose groups of ESL, the shared control groups were equally split and included as independent comparisons to overcome a unit-of-analysis error, according to the instruction of Cochrane's Handbook (20). Data based on the intention-to-treat analysis were extracted for each RCT. The *I*^2^ statistic was also calculated, and an *I*^2^ > 50% reflected significant heterogeneity. Pooled analyses were calculated using a random-effect model with the DerSimonian and Laird approach because this conservative model has incorporated the possible impact of between-study heterogeneity on the outcome (22). Predefined sensitivity and subgroup analyses were conducted to determines the outcomes in adult and child separately, according to the dose of ESL used (22). Publication bias was observed by visual examination of funnel plots, and estimated by the Egger's regression asymmetry test (23). *P*-values < 0.05 were judged as statistically significant. The RevMan (Version 5.1; Cochrane, Oxford, UK) and Stata software (Version 12.0; Stata, College Station, TX) were adopted for statistical analyses.

## Results

### Literature Search

**Figure 1:**
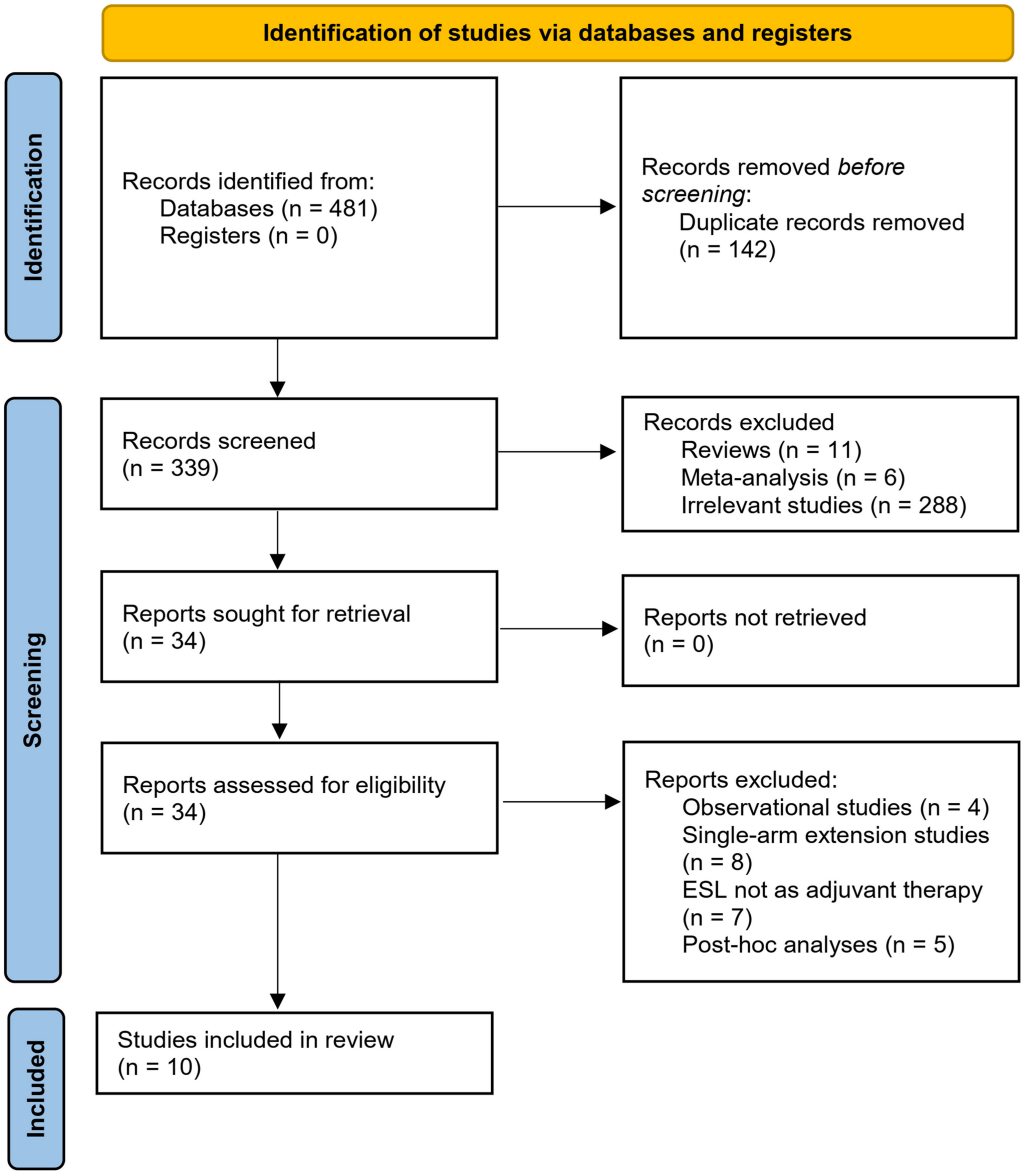
Flowchart of literature search.

The process of database search and study inclusion is shown in Figure 1. In brief, 339 articles were after initial database search and exclusion of the duplicated records. Among them, 305 articles were excluded based on the screening of titles and abstracts, primarily because they were not relevant to the objective of the meta-analysis. Via full-text review, 24 of the remaining 34 articles were further removed, because of the reasons presented in Figure 1, which made 10 RCTs (13–17, 24–28) finally available for the meta-analysis.

### Summary of the Study Features

Table 1 shows the characteristics of the included studies. Overall, eight studies involving 2,179 adults (14, 15, 17, 24–28) and two studies involving 386 children with refractory or uncontrolled FOE contributed to the meta-analysis (13, 16). The studies were published between 2007 and 2021. Seven of them were multicenter RCTs performed in multiple countries (13, 16, 24–28) and three of them were performed in China (14, 15, 17). The sample size of each study varied between 90 and 653. The background treatments included carbamazepine, levetiracetam, or one to three kinds of ASMs. The doses of ESL were 400, 800, or 1,200 mg/d in adults, and 10–30 mg/kg/d in children. The follow-up durations varied from 8–14 weeks. The common types of AE related to included ataxia, dizziness, fatigue, nausea, and somnolence, according to the reports of the included studies.

**Table 1:** Characteristics of included clinical studies.

**Study**	**Locations**	**Design**	**Participants characteristics**	**No. of cases**	**Concomitant treatments**	**ESL regimens**	**Control**	**Treatment durations**
Elger et al. (24)	5 European countries	R, DB, PC	Adults with refractory FOE	143	1–2 ASMs	mean: 800 mg/d	Placebo	12 weeks
Gil-Nagel et al. (26)	Mexico, Portugal and Spain	R, DB, PC	Adults with refractory FOE	252	1–2 ASMs	800 and 1,200 mg/d	Placebo	12 weeks
Elger et al. (25)	11 European countries from Europe and Asia	R, DB, PC	Adults with refractory FOE	402	1–2 ASMs	400, 800, and 1,200 mg/d	Placebo	8 weeks
Ben-Menachem et al. (27)	13 countries from Europe and Asia	R, DB, PC	Adults with refractory FOE	395	1–3 ASMs	400, 800, and 1,200 mg/d	Placebo	14 weeks
Sperling et al. (28)	19 countries from Europe and Asia	R, DB, PC	Adults with uncontrolled FOE	653	1–2 ASMs	800 and 1,200 mg/d	Placebo	12 weeks
Józwiak et al. (13)	4 European countries	R, DB, PC	Children with refractory FOE	123	1–2 ASMs	10–30 mg/kg/d	Placebo	8 weeks
Ma et al. (15)	China	R, DB	Adults with uncontrolled FOE	112	Carbamazepine	800 mg/d	Blank treatment	14 weeks
Fan et al. (14)	China	R, SB	Adults with uncontrolled FOE	132	2–3 ASMs	800 mg/d	Blank treatment	12 weeks
Kirkham et al. (16)	20 countries from Europe and Asia	R, DB, PC	Children with refractory FOE	263	1–2 ASMs	20–30 mg/kg/d	Placebo	12 weeks
Zhang et al. (17)	China	R	Adults with uncontrolled FOE	90	Levetiracetam	800 mg/d	Blank treatment	12 weeks

*R, randomized; DB, double-blind; SB, single-blind; PC, placebo-controlled; ASMs, anti-seizure medications; ESL, Eslicarbazepine Acetate; FOE, focal-onset epilepsy*.

### Data Quality

Table 2 shows the details of study quality evaluation. All of these studies were double-blinded RCTs except for two studies (14, 17). Methods of random sequence generation were reported in three studies (25, 26, 28), and strategies for allocation concealment was reported in one study (28). The overall quality score varied between 3 and 7, reflecting moderate to good study quality.

**Table 2:** Quality evaluation via the Cochrane's risk of bias tool.

**Study**	**Random sequence generation**	**Allocation concealment**	**Blinding of participants**	**Blinding of outcome assessment**	**Incomplete outcome data addressed**	**Selective reporting**	**Other sources of bias**	**Total**
Elger et al. (24)	Unclear	Unclear	Low	Low	Low	Low	Low	5
Gil-Nagel et al. (26)	Low	Unclear	Low	Low	Low	Low	Low	6
Elger et al. (25)	Low	Unclear	Low	Low	Low	Low	Low	6
Ben-Menachem et al. (27)	Unknown	Unclear	Low	Low	Low	Low	Low	5
Sperling et al. (28)	Low	Low	Low	Low	Low	Low	Low	7
Józwiak et al. (13)	Unclear	Unclear	Low	Low	Low	Low	Low	5
Ma et al. (15)	Unclear	Unclear	Low	Low	Low	Low	Low	5
Fan et al. (14)	Unclear	Unclear	Low	Unclear	Low	Low	Low	4
Kirkham et al. (16)	Unclear	Unclear	Low	Low	Low	Low	Low	5
Zhang et al. (17)	Unclear	Unclear	Unclear	Unclear	Low	Low	Low	3

### Efficacy and Tolerability of Adjunctive ESL in Adults With FOE

**Figure 2:**
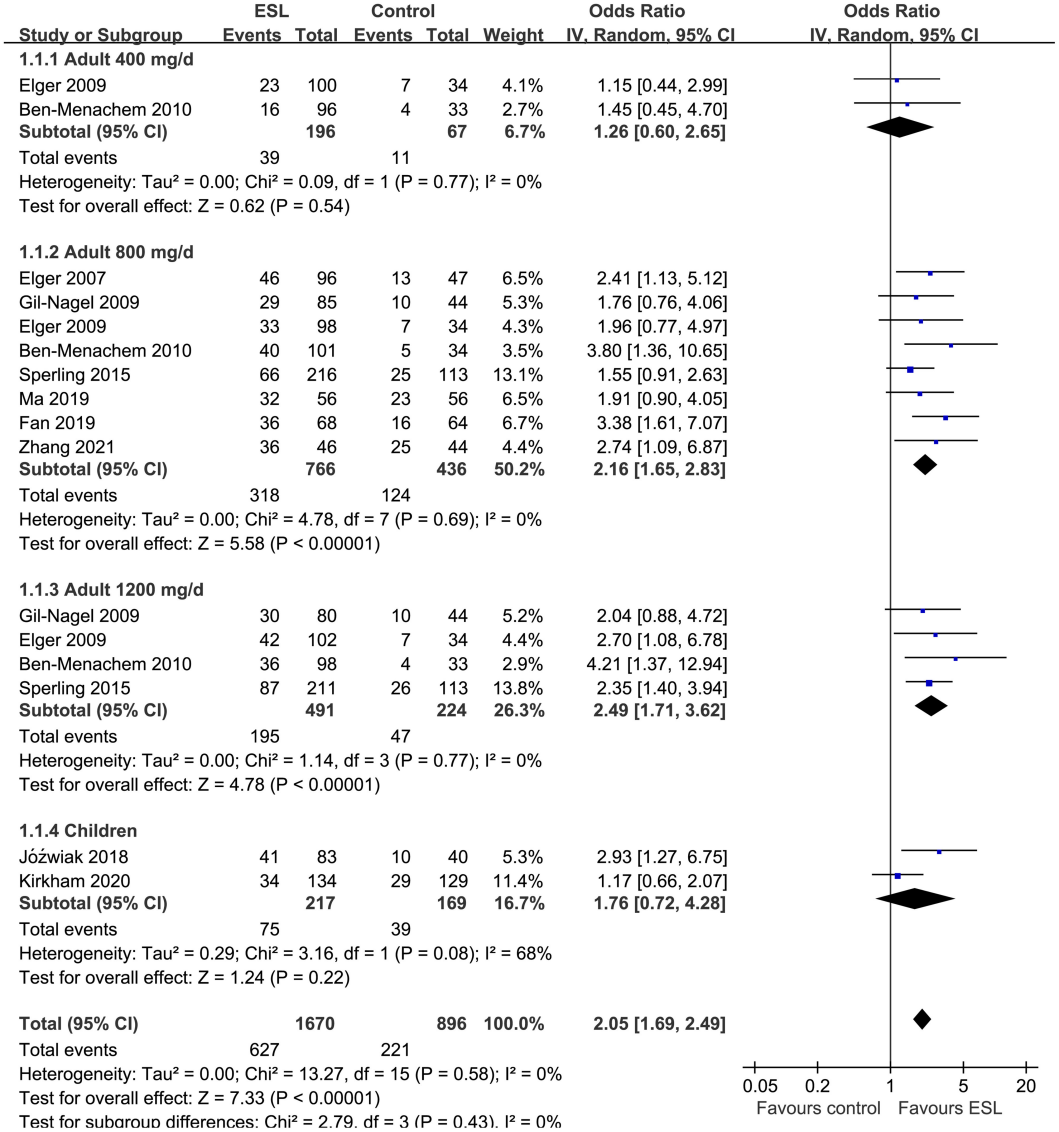
Forest plots for the meta-analysis of the influence of adjunctive ESL on response rate in adults with FOE according to dose of ESL and in children with all doses.

**Figure 3:**
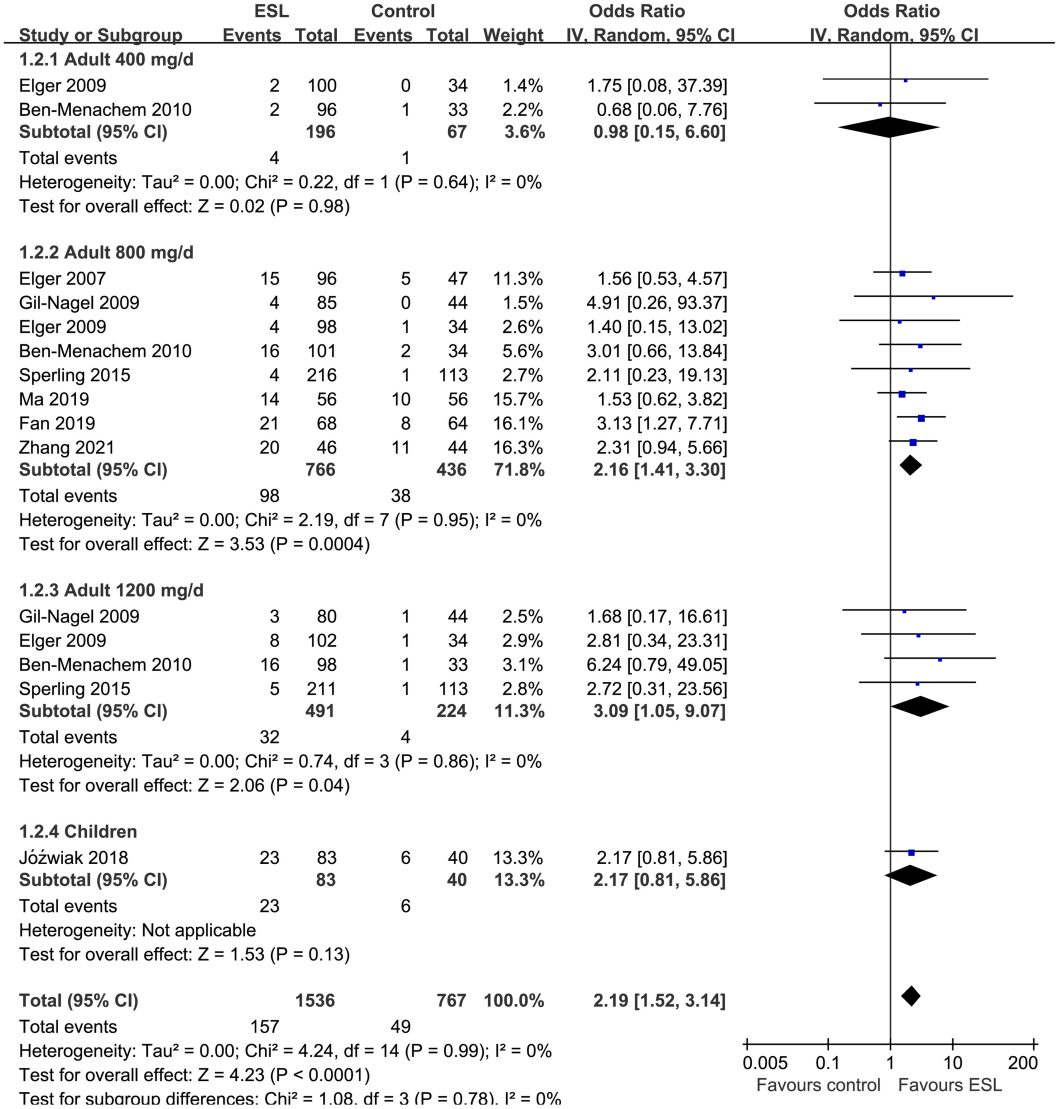
Forest plots for the meta-analysis of the influence of adjunctive ESL on effective rate in adults with FOE according to dose of ESL and in children with all doses.

**Figure 4:**
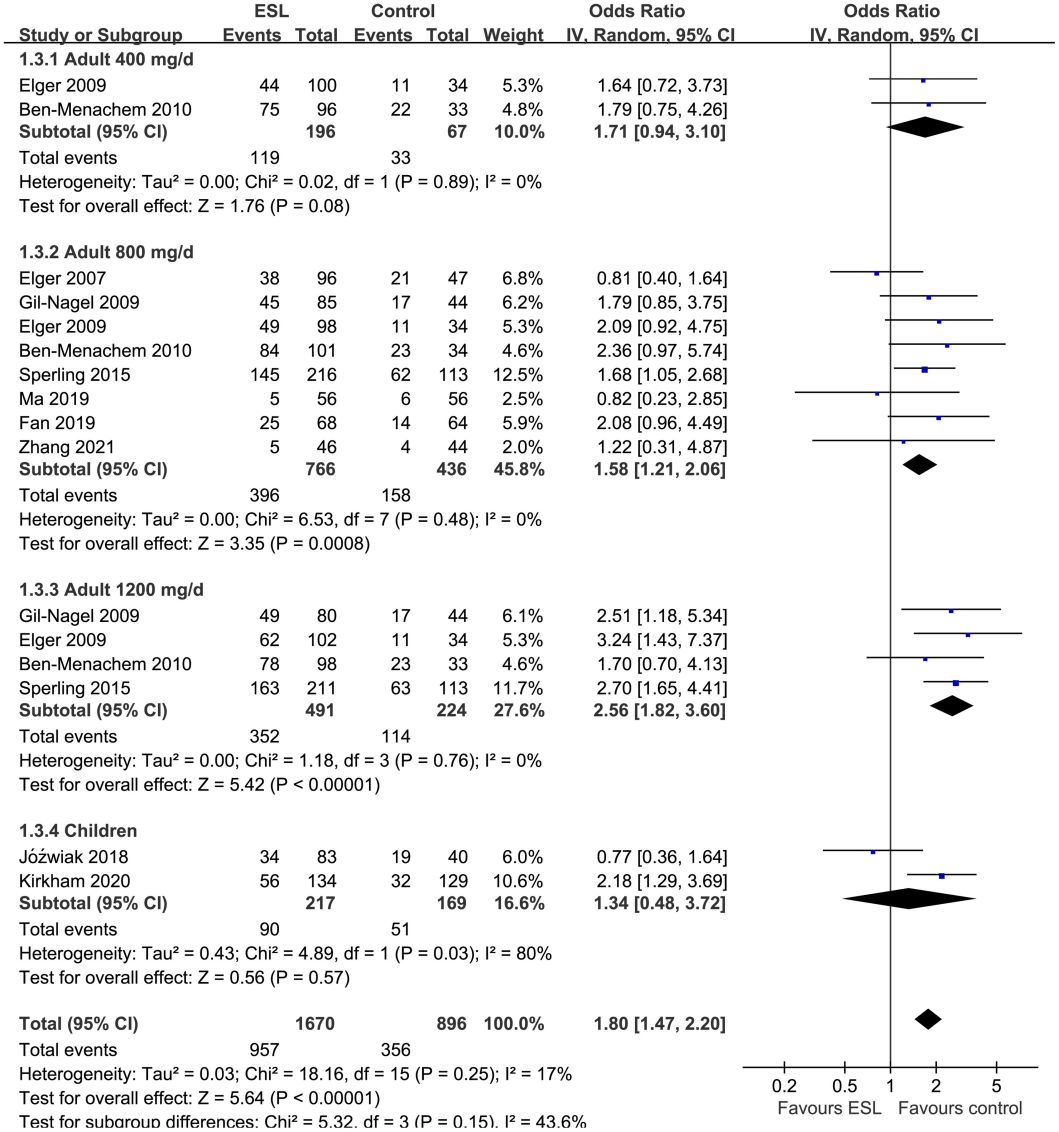
Forest plots for the meta-analysis of the influence of adjunctive ESL on the incidence of overall AE in adults with FOE according to dose of ESL and in children with all doses.

**Figure 5:**
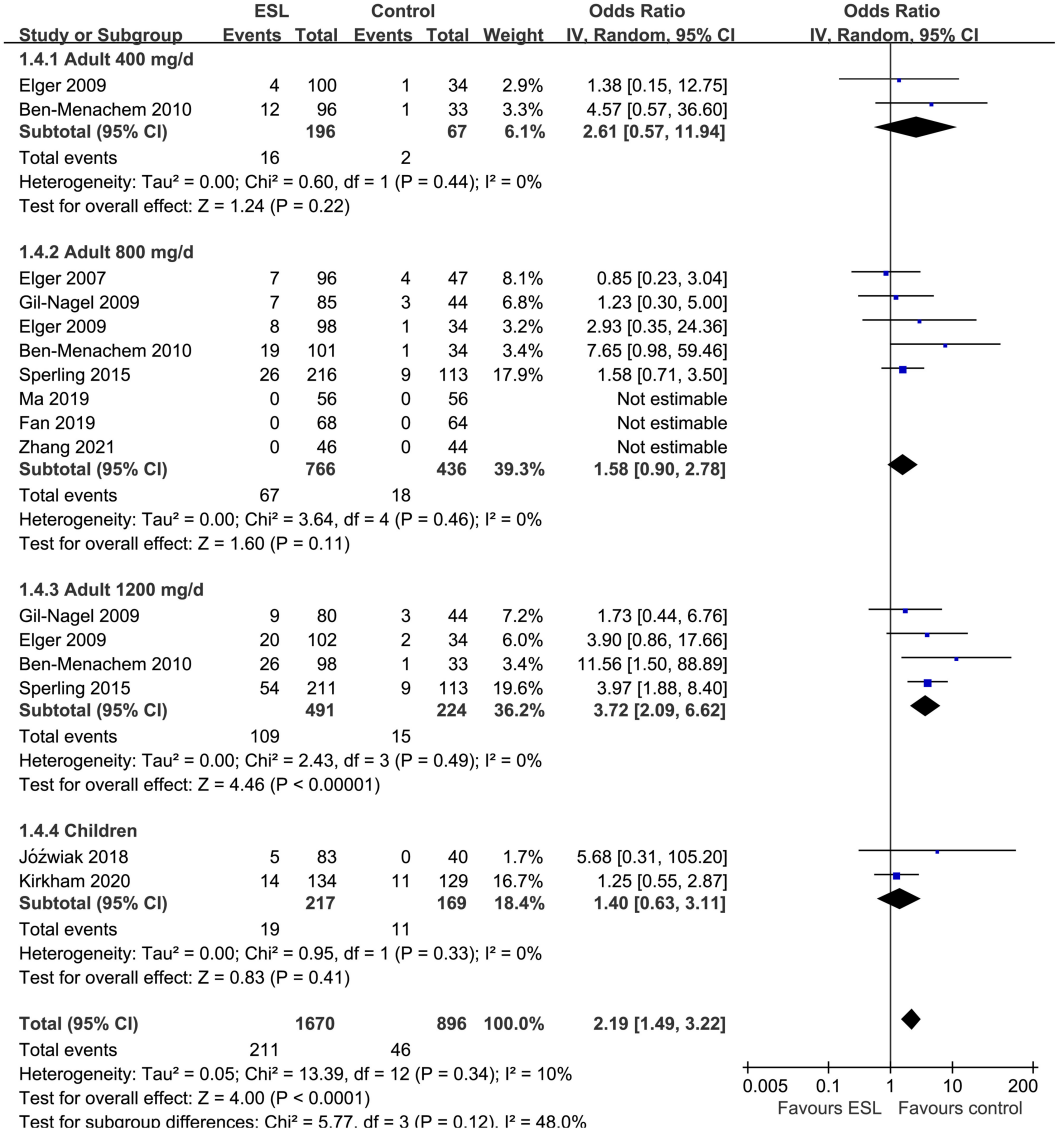
Forest plots for the meta-analysis of the influence of adjunctive ESL on the incidence of severe AE leading to drug discontinuation in adults with FOE according to dose of ESL and in children with all doses.

Pooled results showed that ESL 400 mg/d did not significantly improve the response rate (OR 1.26, 95% CI 0.60–2.65, *p* = 0.54, *I*^2^ = 0%; Figure 2) or the effective rate (OR 0.98, 95% CI 0.15 to 6.60, *p* = 0.98, *I*^2^ = 0%; Figure 3) in adults of FOE. Moreover, the incidence of overall AE (OR 1.71, *p* = 0.08; Figure 4) or severe AE (OR: 2.61, *p* = 0.22; Figure 5) were also not significantly affected. Adjunctive treatment with ESL 800 mg/d was associated with improved response rate (OR 2.16, CI: 1.65–2.83, *p* < 0.001; *I*^2^ = 0%; Figure 2) and effective rate (OR 2.16, 95% CI: 1.41–3.30, *p* < 0.001; *I*^2^ = 0%; Figure 3). Although the overall AE was increased in people with ESL (OR 1.58, 95% CI 1.21–2.06, *I*^2^ = 0%; Figure 4), the incidence of severe AE leading to drug discontinuation was not significantly affected (OR 1.58, 95% CI: 0.90–2.78, *p* = 0.11, *I*^2^ = 0%; Figure 5). Since most of the included studies applied 12-week treatment duration, sensitivity analyses limited to studies with ESL 800 mg/d for 12 week (14, 17, 24, 26, 28) were also performed. The results showed that adjunctive treatment with ESL 800 mg/d for 12 week was associated with improved response rate (OR 2.12, 95% CI: 1.54–2.92, *p* < 0.001, *I*^2^ = 0%) and the effective rate (OR 2.37, 95% CI: 1.41–4.01, *p* = 0.001, *I*^2^ = 0%) in adults, without increasing the risk of severe AE (OR 1.31, 95% CI: 0.71–2.41, *p* = 0.39, *I*^2^ = 0%). In addition, ESL 1,200 mg/d significantly improved response rate (OR 2.49, *p* < 0.001, *I*^2^ = 0%; Figure 2) and effective rate (OR 3.09, *p* = 0.04, *I*^2^ = 0%; Figure 3), but also significantly increased the overall AE (OR 2.56, *p* < 0.001, *I*^2^ = 0%; Figure 4) and the severe AE (OR 3.72, *p* < 0.001, *I*^2^ = 0%; Figure 5).

### Efficacy and Tolerability of Adjunctive ESL in Children With FOE

For children, ESL did not significantly improve the response rate (OR 1.76, 95% CI 0.72–4.28, *p* = 0.22, *I*^2^ = 68%; Figure 2), the effective rate (OR 2.17, 95% CI 0.81–5.86, *p* = 0.13; Figure 3), and the incidence of overall (OR: 1.34, *p* = 0.57; Figure 4) or severe AE (OR: 1.40, *p* = 0.41; Figure 5).

### Publication Bias

**Figure 6:**
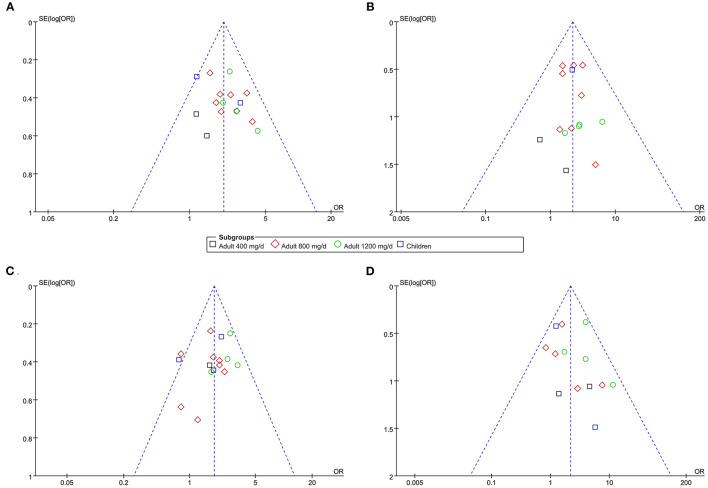
Funnel plots for the publication bias within the meta-analyses. **(A)** funnel plots for the meta-analysis of response rate. **(B)** funnel plots for the meta-analysis of effective rate. **(C)** funnel plots for the meta-analysis of overall AE. **(D)** funnel plots for the meta-analysis of severe AE.

The funnel plots for the meta-analyses of effective rate, response rate, overall AE, and severe AE were symmetrical, suggesting low-risk of publication bias (Figures 6A–D). Egger's regression test also showed low risk of publication bias (P for Egger's regression test all >0.10).

## Discussion

Evaluating the potential efficacy and safety of adjunctive treatment for people with refractory FOE is clinically important, in view of the adverse influences of recurrent episodes of seizures on quality of life in these people. Results of the current meta-analysis, by integrating the evidence from available RCTs, showed that ESL 800 mg/d as adjunctive treatment is effective for reducing the frequency of seizure in adults with FOE, without increasing of severe AE leading to drug discontinuation. However, for children with FOE, no significant improvement in the response rate or the effective rate could be observed after adds-on therapy with ESL. Taken together, these finding suggested that ESL 800 mg/d is effective and well-tolerated as adjunctive treatment for adults with FOE. Efficacy of ESL in children with FOE should be further evaluated.

Results of our meta-analysis suggested that in adults with FOE, the efficacy of ESL in reducing the frequency of episodes of seizure is related to the dose of the drug. Additional use of ESL significantly increased the response and effective rates in adults with FOE at doses of 800 and 1,200 mg/d, but not for a relative low dose of 400 mg/d. The dose-dependent anti-seizure efficacy of ESL was also observed in several preclinical studies. An early study in amygdala kindling model of temporal lobe epilepsy in mice showed that ESL dose-dependently increased threshold of focal seizure (29). Using a mouse model of KCNQ2-related epilepsy, a recent study showed that ESL could dose-dependently exert the protection efficacy from seizure (30). However, it has to be mentioned that the result of inefficacy of the 400 mg dosage cannot be generalized because in special populations as in elderly people with epilepsy, ESL of 400 mg dosage can be effective in controlling seizures (31, 32). As for the mechanism underlying the possible therapeutic efficacy of additional ESL on FOE, some studies proposed other mechanisms besides the direct blockage of ESL on voltage-gated sodium channels may be involved. A recent study in rat model of epilepsy caused by chronic pilocarpine treatment showed that the influence of ESL on the level of neuronal networks could not be fully explained by its pharmacological effect on voltage-dependent sodium channels, and may be related to the influence on the plasticity of inhibitory circuits (33). Moreover, another study showed that ESL therapy could induce a significant enhancement of brain activity and connectivity, and the post-ESL connectivity profile of people with epilepsy was similar to the one of healthy controls (34). Studies are warranted for further investigation.

Since an important consideration besides the efficacy in determination of the ASMs in clinical use is the safety of the drug, it is also pivotal to evaluate the tolerability of different doses of ESL for adults with FOE. Although both ESL at 800 and 1,200 mg/d were associated with increased risks of overall AEs, ESL at 1,200 mg/d also significantly increased the risk of severe AEs leading to drug discontinuation, but not for ESL at 800 mg/d. This is consistent with the results of a recent meta-analysis, which showed that the incidence of severity of AEs related to ESL was dose-dependent (35). Balancing the efficacy and tolerability of the drug, ESL at 800 mg/d is effective and well-tolerated as adjunctive treatment for adults with FOE, which is consistent with the current recommendations. Consistently, several long-term extension studies also support the long-term efficacy and tolerability of ESL for adults of FOE (36, 37), even with ESL mono therapy (38, 39). Besides, it has been also suggested by some retrospective cohort studies that early adjunctive treatment with ESL in adults of FOE may be more cost effective than later add-on of the drug (40, 41). All of the above evidence suggests early use of ESL as an add-on treatment for adults with refractory FOE.

Results of our meta-analysis by including two studies showed that adjunctive treatment with ESL did not seem to significantly affect the response or effective rates and incidence of AEs in children with refractory FOE. A previous meta-analysis including two RCTs was published in 2018 to evaluate the efficacy and safety of ESL in children with FOE (42). Although both the meta-analyses showed that ESL was well-tolerated in children with FOE, the efficacy outcomes were different between the previous and the current meta-analysis. The previous meta-analysis, which also included the two RCTs of children with FOE, showed that treatment with ESL was associated with reduced seizure frequency compared to the baseline level (42). However, the outcomes of the response and effective rates were not observed in the previous meta-analysis (42). Our meta-analysis, on the contrary, showed that ESL did not significantly improve the response rate or the effective rate in children, suggesting the uncertainty of the efficacy of ESL in children with FOE. Results of the findings from both the meta-analyses should be interpreted caution because of the limited datasets and significant between-study heterogeneity. Further studies are needed for clarification in this circumstance.

The strengths of the meta-analysis include up-to-date literature search, detailed analysis according to the age group and dosages of ESL, and comprehensive analysis with both the efficacy and safety outcomes. However, there are also some limitations of the meta-analysis which should be paid attention to. First, datasets involved in some subgroups were limited, such as those of ESL at 400 mg for adults and ESL for children with FOE. Results of the related analyses should be investigated in the future. Second, the follow-up durations were relatively short, varying between 8 and 14 weeks. Although several observational studies have suggested the long-term efficacy and tolerability of ESL in adults with FOE, long-term clinical trials are still needed for the confirmation. Finally, comparative efficacy and safety between ESL and other potential adds-on therapies in people with FOE remain to be determined. Although a recent network meta-analysis showed that the efficacy of ESL was inferior to cenobamate and the tolerability of ESL was inferior to brivaracetam or lacosamide (43), the results were based on indirect comparison in network meta-analysis. Further direct head-to-head clinical studies should be performed to evaluate the efficacy and tolerability of ESL as compared to other third-generation ASMs for people with of FOE.

To sum up, results of the current meta-analysis indicated that ESL 800 mg/d is effective and well-tolerated as adjunctive treatment for adults with refractory FOE. Efficacy and tolerability of ESL as adds-on therapy in children with refractory FOE should be further evaluated in future studies.

## Data Availability Statement

The original contributions presented in the study are included in the article/supplementary material, further inquiries can be directed to the corresponding author/s.

## Author Contributions

YF and ZS conceived the study and interpreted the results. YF and RS performed database search, literature review, quality evaluation, and statistical analyses. YF drafted the manuscript. All authors reviewed the manuscript and approved the submission.

## Funding

This study was supported by the New Xiangya Talent Project of the Third Xiangya Hospital of Central South University (grant number: 20170310).

## Conflict of Interest

The authors declare that the research was conducted in the absence of any commercial or financial relationships that could be construed as a potential conflict of interest.

## Publisher's Note

All claims expressed in this article are solely those of the authors and do not necessarily represent those of their affiliated organizations, or those of the publisher, the editors and the reviewers. Any product that may be evaluated in this article, or claim that may be made by its manufacturer, is not guaranteed or endorsed by the publisher.
